# Overview of Cattle Diseases Listed Under Category C, D or E in the Animal Health Law for Which Control Programmes Are in Place Within Europe

**DOI:** 10.3389/fvets.2021.688078

**Published:** 2021-07-30

**Authors:** Jaka Jakob Hodnik, Žaklin Acinger-Rogić, Mentor Alishani, Tiina Autio, Ana Balseiro, John Berezowski, Luís Pedro Carmo, Ilias Chaligiannis, Beate Conrady, Lina Costa, Iskra Cvetkovikj, Ivana Davidov, Marc Dispas, Igor Djadjovski, Elsa Leclerc Duarte, Céline Faverjon, Christine Fourichon, Jenny Frössling, Anton Gerilovych, Jörn Gethmann, Jacinto Gomes, David Graham, Maria Guelbenzu, George J. Gunn, Madeleine K. Henry, Petter Hopp, Hans Houe, Elena Irimia, Jožica Ježek, Ramon A. Juste, Emmanouil Kalaitzakis, Jasmeet Kaler, Selcuk Kaplan, Polychronis Kostoulas, Kaspars Kovalenko, Nada Kneževič, Tanja Knific, Xhelil Koleci, Aurélien Madouasse, Alvydas Malakauskas, Rene Mandelik, Eleftherios Meletis, Madalina Mincu, Kerli Mõtus, Violeta Muñoz-Gómez, Mihaela Niculae, Jelena Nikitović, Matjaž Ocepek, Marie Tangen-Opsal, László Ózsvári, Dimitrios Papadopoulos, Theofilos Papadopoulos, Sinikka Pelkonen, Miroslaw Pawel Polak, Nicola Pozzato, Eglé Rapaliuté, Stefaan Ribbens, João Niza-Ribeiro, Franz-Ferdinand Roch, Liza Rosenbaum Nielsen, Jose Luis Saez, Søren Saxmose Nielsen, Gerdien van Schaik, Ebba Schwan, Blagica Sekovska, Jože Starič, Sam Strain, Petr Šatran, Sabina Šerić-Haračić, Lena-Mari Tamminen, Hans-Hermann Thulke, Ivan Toplak, Erja Tuunainen, Sharon Verner, Štefan Vilček, Ramazan Yildiz, Inge M. G. A. Santman-Berends

**Affiliations:** ^1^Clinic for Reproduction and Large Animals – Section for Ruminants, Veterinary Faculty, University of Ljubljana, Ljubljana, Slovenia; ^2^Veterinary and Food Safety Directorate, Ministry of Agriculture, Zagreb, Croatia; ^3^Department of Veterinary Medicine, Faculty of Agriculture and Veterinary, University of Prishtina “Hasan Prishtina”, Prishtina, Albania; ^4^Finnish Food Authority, Veterinary Bacteriology and Pathology Unit, Kuopio, Finland; ^5^Animal Health Department, University of León, León, Spain; ^6^Animal Health Department, Instituto de Ganadería de Montaña Consejo Superior de Investigaciones Científicas-University of León, León, Spain; ^7^Veterinary Public Health Institute, Vetsuisse, University of Bern, Bern, Switzerland; ^8^School of Veterinary Medicine, Aristotle University of Thessaloniki, Thessaloniki, Greece; ^9^Department of Veterinary and Animal Sciences, Faculty of Health and Medical Sciences, University of Copenhagen, Copenhagen, Denmark; ^10^Complexity Science Hub Vienna, Vienna, Austria; ^11^Department of Agrarian and Veterinary Sciences, Agrarian School of Elvas, Polytechnic Institute of Portalegre, Portalegre, Portugal; ^12^Faculty of Veterinary Medicine in Skopje, Ss Cyril and Methodius University in Skopje, Skopje, Macedonia; ^13^Faculty of Agriculture, University of Novi Sad, Novi Sad, Serbia; ^14^Sciensano, Brussels, Belgium; ^15^Departamento de Medicina Veterinária, Mediterranean Institute for Agriculture, Environment and Development, Universidade de Évora, Évora, Portugal; ^16^Ausvet Europe, Lyon, France; ^17^INRAE, Oniris, BIOEPAR, Nantes, France; ^18^Department of Disease Control and Epidemiology, National Veterinary Institute (SVA), Uppsala, Sweden; ^19^Department of Animal Environment and Health, Swedish University of Agricultural Sciences, Skara, Sweden; ^20^National Scientific Centre, Institute for Experimental and Clinical Veterinary Medicine, Kharkiv, Ukraine; ^21^Friedrich-Loeffler-Institut, Federal Research Institute for Animal Health, Institute of Epidemiology, Greifswald, Germany; ^22^Animal Health and Production Unit, National Institute for Agrarian and Veterinary Research, Oeiras, Portugal; ^23^Animal Health Ireland, Carrick on Shannon, Ireland; ^24^Epidemiology Research Unit, Department of Veterinary and Animal Science, Northern Faculty, Scotland's Rural College, Inverness, United Kingdom; ^25^Section of Epidemiology, Norwegian Veterinary Institute (NVI), Oslo, Norway; ^26^Research and Development Institute for Bovine Balotesti, Balotesti, Romania; ^27^Department of Animal Health, NEIKER-Basque Institute for Agricultural Research and Development, Basque Research and Technology Alliance, Derio, Spain; ^28^Clinic of Farm Animals, Veterinary Faculty, Aristotle University Thessaloniki, Thessaloniki, Greece; ^29^School of Veterinary Medicine and Science, University of Nottingham, Nottingham, United Kingdom; ^30^Department of Genetics, Faculty of Veterinary Medicine, Tekirdag Namik Kemal University, Tekirdag, Turkey; ^31^Laboratory of Epidemiology, Faculty of Public and One (Integrated) Health, School of Health Sciences, University of Thessaly, Karditsa, Greece; ^32^Faculty of Veterinary Medicine, Latvia University of Lifesciences and Technologies, Jelgava, Latvia; ^33^Podravka Food Industry, Research and Development, Koprivnica, Croatia; ^34^Veterinary Faculty, Institute of Food Safety, Feed and Environment, University of Ljubljana, Ljubljana, Slovenia; ^35^Department of Veterinary Public Health, Faculty of Veterinary Medicine, Agricultural University of Tirana, Tirana, Albania; ^36^Department of Veterinary Pathobiology, Lithuanian University of Health Sciences, Veterinary Academy, Kaunas, Lithuania; ^37^Department of Epizootiology, Parasitology and Protection of One Health, University of Veterinary Medicine and Pharmacy, Kosice, Slovakia; ^38^Institute of Veterinary Medicine and Animal Sciences, Estonian University of Life Sciences, Tartu, Estonia; ^39^Section of Epidemiology, Vetsuisse Faculty, University of Zürich, Zürich, Switzerland; ^40^Faculty of Veterinary Medicine, University of Agricultural Sciences and Veterinary Medicine Cluj-Napoca, Cluj-Napoca, Romania; ^41^Institute for Genetic Resources, University of Banja Luka, Banja Luka, Bosnia and Herzegovina; ^42^Veterinary Faculty, National Veterinary Institute, University of Ljubljana, Ljubljana, Slovenia; ^43^Norwegian Food Safety Authority, Oslo, Norway; ^44^Department of Veterinary Forensics and Economics, University of Veterinary Medicine Budapest, Budapest, Hungary; ^45^Department of Microbiology, Faculty of Veterinary Medicine, Aristoteles University of Thessaloniki, Thessaloniki, Greece; ^46^National Veterinary Research Institute, Pulawy, Poland; ^47^Laboratorio di Medicina Forense Veterinaria, Struttura Complessa Territoriale 1 - Verona e Vicenza, Istituto Zooprofilattico Sperimentale Delle Venezie, Vicenza, Italy; ^48^Animal Health Care Flanders, Torhout, Belgium; ^49^Department of Population Studies, Institute of Biomedical Sciences Abel Salazar, University of Porto, Porto, Portugal; ^50^Unit of Food Microbiology, Institute for Food Safety, Food Technology and Veterinary Public Health, University of Veterinary Medicine Vienna, Vienna, Austria; ^51^Ministry of Agriculture, Fisheries and Food, Madrid, Spain; ^52^Department of Population Health Sciences, Faculty of Veterinary Medicine, Utrecht University, Utrecht, Netherlands; ^53^Royal GD, Deventer, Netherlands; ^54^Farm and Animal Health, Uppsala, Sweden; ^55^Animal Health and Welfare Northern Ireland, Dungannon, United Kingdom; ^56^State Veterinary Administration, Prague, Czechia; ^57^Animal Health Economics Department, Veterinary Faculty of the University of Sarajevo, Sarajevo, Bosnia and Herzegovina; ^58^Swedish University of Agricultural Sciences, Uppsala, Sweden; ^59^Department of Ecological Modelling, Helmholtz Centre for Environmental Research – UFZ, Leipzig, Germany; ^60^Department of Virology, Veterinary Faculty, Institute of Microbiology and Parasitology, University of Ljubljana, Ljubljana, Slovenia; ^61^Animal Health ETT, Seinäjoki, Finland; ^62^Department of Internal Medicine, Faculty of Veterinary Medicine, Burdur Mehmet Akif Ersoy University, Burdur, Turkey

**Keywords:** disease control, SOUND control, control programmes, Europe, cattle, output-based standards

## Abstract

The COST action “Standardising output-based surveillance to control non-regulated diseases of cattle in the European Union (SOUND control),” aims to harmonise the results of surveillance and control programmes (CPs) for selected cattle diseases to facilitate safe trade and improve overall control of cattle infectious diseases. In this paper we aimed to provide an overview on the diversity of control for these diseases in Europe. A selected cattle disease was defined as an infectious disease of cattle with no or limited control at EU level, which is not included in the European Union Animal health law Categories A or B under Commission Implementing Regulation (EU) 2020/2002. A CP was defined as surveillance and/or intervention strategies designed to lower the incidence, prevalence, mortality or prove freedom from a specific disease in a region or country. Passive surveillance, and active surveillance of breeding bulls under Council Directive 88/407/EEC were not considered as CPs. A questionnaire was designed to obtain country-specific information about CPs for each disease. Animal health experts from 33 European countries completed the questionnaire. Overall, there are 23 diseases for which a CP exists in one or more of the countries studied. The diseases for which CPs exist in the highest number of countries are enzootic bovine leukosis, bluetongue, infectious bovine rhinotracheitis, bovine viral diarrhoea and anthrax (CPs reported by between 16 and 31 countries). Every participating country has on average, 6 CPs (min–max: 1–13) in place. Most programmes are implemented at a national level (86%) and are applied to both dairy and non-dairy cattle (75%). Approximately one-third of the CPs are voluntary, and the funding structure is divided between government and private resources. Countries that have eradicated diseases like enzootic bovine leukosis, bluetongue, infectious bovine rhinotracheitis and bovine viral diarrhoea have implemented CPs for other diseases to further improve the health status of cattle in their country. The control of the selected cattle diseases is very heterogenous in Europe. Therefore, the standardising of the outputs of these programmes to enable comparison represents a challenge.

## Introduction

Animal disease control programmes (CPs) provide benefits for animals, farmers, the industry and consumers, because they increase animal health and welfare, decrease antibiotic use and in the case of zoonotic diseases improve the safety of animal products. CPs reduce direct and indirect disease losses (1). Their implementation involves associated costs for testing and administrative work; however, these costs are generally considered to be outweighed by the benefits.

The control of some cattle diseases in the European Union (EU) is currently founded on input-based standards, by which the EU prescribes all the activities a country must implement to reach the desired output, confidence of freedom from infection or disease. However, there is an international trend to move to output-based standards, which do not prescribe how the end goal (confidence of freedom from infection or disease) must be achieved and allows for country specific control or eradication measures (2). The move to output-based standards would allow for safe trade of cattle between territories that have achieved the desired confidence of freedom, without additional costs for testing of individual animals (3). Additionally, because EU member states are not allowed to set trade restrictions on intracommunity trade for selected cattle diseases, countries that have achieved freedom from specific diseases are at risk of their reintroduction with imported animals. Therefore, available information on the current control and disease status in each country would greatly aid farmers and authorities when considering the risk of importing live cattle from these countries.

“Standardising output-based surveillance to control non-regulated diseases of cattle in the European Union” (SOUND control) is a COST action (CA 17110) aiming to harmonise the results of surveillance and control programmes for selected cattle diseases to facilitate safe trade, and to reduce the economic impact and improve overall control of infectious cattle diseases. This COST action connects more than 100 members from different fields (including veterinarians, epidemiologists, economists, statisticians, sociologists and policy makers) from 33 European countries. An overview of the project was published by Costa et al. (1). The first working group within the action aims to identify cattle diseases with no or limited regulation at European level for which CPs are in place and to describe the characteristics of these CPs. To obtain this information clear definitions of CPs and disease statuses had to be set to allow the comparisons of the heterogeneous CPs.

Similar evaluations have been undertaken for bovine viral diarrhoea and paratuberculosis (3, 4), but these studies were limited to only one disease. In 2017, the European Food Safety Authority (EFSA) published information on EU countries' disease statuses for certain cattle diseases (5–14); however, different definitions were used and not all of the selected diseases were covered. Furthermore, not all European countries were included and some of the data are now outdated.

This paper aims to provide a comprehensive overview of the current (end of 2020) disease status and control efforts for selected cattle diseases with no or limited regulation at European level, for all 33 European countries that participate in the SOUND control project in 2020. To the best of the authors' knowledge, this is the first overview of cattle disease CPs in Europe incorporating so wide a range of diseases and representing so many countries.

## Materials and Methods

A questionnaire was designed to collect disease and CP information from all participating countries. To allow for comparison of heterogeneous CPs between countries, it was necessary to ensure definitions were clear and an exhaustive list of diseases for which CPs might exist was included. The questionnaire was developed through an iterative process with input from all members of the COST action. Action members from all participating countries (33 in total) were asked to complete the final survey.

### Definitions

The definitions for the survey were agreed upon at a series of meetings involving members of all countries participating in SOUND control. First, the definition of a selected cattle disease had to be clarified. Initially, such diseases were defined as diseases with no or limited regulation at EU level. However, given the adoption of the new Animal Health Law (AHL) (15), most cattle diseases were categorised at some level and the definition of selected cattle diseases had to be aligned with the changed law. Additionally, definitions had to be determined for a disease CP and a country disease status. The final selected definitions were:

*Disease* means the occurrence of infections and infestations in animals, with or without clinical or pathological manifestations, caused by one or more disease agents (15).

*Selected cattle diseases* are defined as infectious cattle diseases not included in the AHL category A or B (15), but for which there are CPs in place in the COST action member countries. This definition also includes diseases for which eradication has been achieved and surveillance is ongoing.

*A CP* was defined as surveillance and/or intervention strategies designed to lower the incidence, prevalence, mortality or prove freedom from a specific disease in a region or country. Passive surveillance alone is excluded as a CP, as it does not provide adequate information on the current disease prevalence in the country to facilitate safe trade without additional testing. An exception was made for anthrax due to the peracute nature of the disease and the long persistence of spores in the ground, if countries had additional long-term control measures (e.g., vaccination) in place. Surveillance of breeding bulls under the Council Directive 88/407/EEC (16) is also excluded as a specific CP, because this action is regulated by the EU and therefore implemented in all EU member states. A CP is implemented on a regional or national level. For the purposes of this survey, a CP had to include multiple herds, be run by an organisation or government, and the herd status of participating farms should be known both centrally by that organisation and by the respective farmers.

*Region*s are politically defined territories defined by each country (states, principalities etc.).

*Dairy cattle* are cattle used for milk production.

*Non-dairy cattle* are all cattle not used for milk production (suckler cows, fattening bulls, veal calves, etc.).

The different disease statuses that could be chosen for the country specific disease status were specified after thorough discussions with the members and are described in Table 1.

**Table 1:** Definitions for terms used to describe type of control programme (CP) and country status for disease in this survey of CPs among countries in Europe.

**Definition**	**Description**
Control	It is the reduction of the morbidity and mortality from disease. It is a general term embracing all measures intended to interfere with the unrestrained occurrence of disease, whatever its cause.
Eradication	Most commonly in veterinary medicine, eradication refers to the regional extinction of an infectious agent. However, it could also be applied at individual herd level.
Surveillance	The collection, collation, analysis and dissemination of data; a type of observational study that involves continuous monitoring of disease occurrence within a population.
Endemic	Endemic is an adjective used in two senses: 1. the usual frequency of occurrence of a disease in a population; 2. the constant presence of a disease in a population.
Sporadic	Is the type of disease that presents irregularly and haphazardly. This implies that appropriate circumstances have occurred locally, producing small, localised outbreaks.
Officially free	Means that a country is officially recognised as free by EU laws.
Perceived free	Means the country does not have an officially free status because it is not available or that they have not had disease cases in the past few years and believe they are free of the disease.
Unknown	Means that the countries (or the members from the country) do not know if they have a CP and/or their disease status.

*The definitions are based on Thrusfield and Christley (17)*.

**Figure 1:**
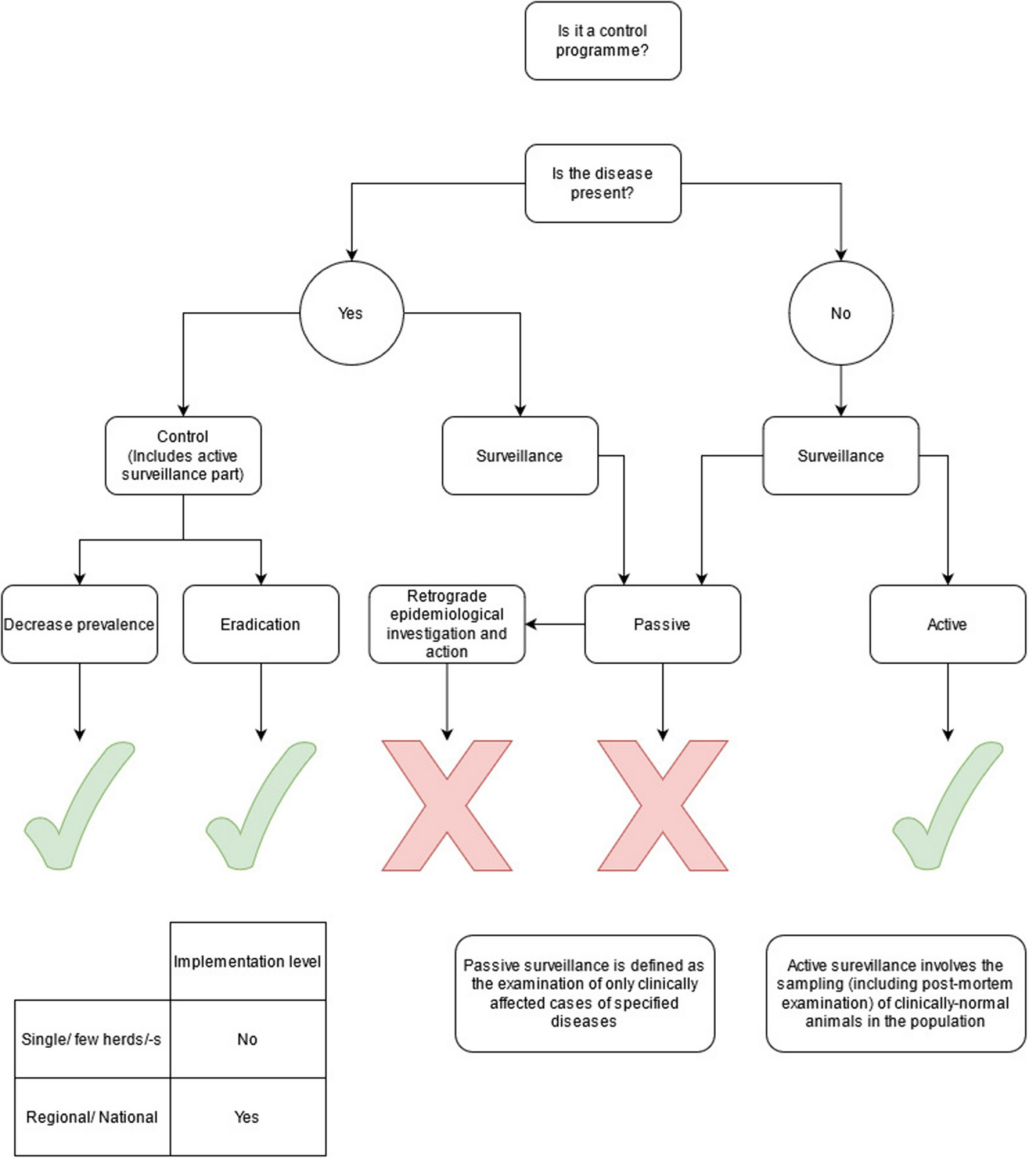
Flowchart giving inclusion and exclusion criteria for control programmes included in the survey.

To help the members determine whether collective actions in their country could be defined as a CP or not, a scheme was developed to support a standardised and objective decision-making (Figure 1). Note that CPs in countries where the disease was still present were considered as having an active surveillance component as part of the CP (to decrease prevalence or eradicate the disease); therefore, active surveillance alone was not an option in these circumstances.

### Development of the Questionnaire on Existing Control Programmes for Selected Cattle Diseases

After agreeing the definitions, a preliminary questionnaire was developed to establish which cattle diseases are currently controlled in SOUND control member countries. Eleven diseases were included, with the option to add additional diseases where a relevant CP existed in a member country. Members from each country had to provide information on the existence of a CP, type of cattle, type of programme (voluntary/compulsory, regional/national and control/eradication/surveillance), funding source, whether there were additional EU guarantees in place and the disease status in the country. Additional EU guarantees referred to restriction in trade of live cattle to the countries that had a superior health status based on EU legislation. The questions were discussed within the consortium and further clarified if needed. Thereafter the questionnaire was sent out to all members. The information on existing CPs in action member countries was collected. During this exercise, more issues arose due to varied interpretation of certain questions by individuals completing the survey. Therefore, the first results and discussion points were presented to the whole group during another meeting and definitions were refined. Based on the information gathered, a list was compiled, comprising 23 diseases that were controlled by at least one country: anthrax (*Bacillus anthracis*), Aujeszky's disease, bluetongue (BT), bovine coronavirus infection, bovine digital dermatitis, bovine genital campylobacteriosis (*Campylobacter fetus* subsp. *venerealis*), bovine respiratory disease (bovine respiratory syncytial virus), bovine viral diarrhoea (BVD), enzootic bovine leukosis (EBL), epizootic haemorrhagic disease, infectious bovine rhinotracheitis (IBR), leptospirosis (Leptospira Hardjo), liver fluke, mycoplasmosis (*Mycoplasma bovis*), contagious bovine pleuropneumonia (*Mycoplasma mycoides subsp. mycoides SC*), neosporosis, paratuberculosis, Q-fever (*Coxiella burnetti*), salmonellosis, staphylococcal infection (*Staphylococcus aureus*), streptococcal infection (*Streptococcus agalactiae*), ringworm (*Trichophyton verrucosum*) and trichomonosis (*Tritrichomonas foetus*).

This resulted in a new and improved version of the questionnaire being circulated to all members in August 2019, with responses provided before the end of 2020. An extensive time period was used in order to obtain information from as many countries as possible. Only one questionnaire was filled in per country. Members obtained the data from their national veterinary authorities' databases, annual country World Animal Health Information System reports, their own research work and opinions of relevant experts. The members had the option to update the information before and during the writing of this manuscript if the situation in their country changed. The members were requested to check the validity of the information when drafting the final version of this manuscript. Therefore, this manuscript provides information that was current at the end of 2020. The following information was requested for each disease: (i) If there was a CP in place for this disease (Yes or No), (ii) The type of cattle that the CP applied to (e.g., dairy, non-dairy, breeding bulls, all types of cattle), (iii) If the CP was voluntary or compulsory, (iv) If the CP was regional or national in terms of coverage, (v) What was the funding arrangement for the CP (e.g., private or government or co-funded between private and public), (vi) Type of CP (Surveillance, Control, Eradication, with possible combinations), (vii) If there were additional EU guarantees for cattle trade in place for that disease (Yes, No and not applicable), (viii) What was the country status for the disease [e.g., officially free (EU level), perceived free, endemic, sporadic, never studied, unknown], (ix) Last occurrence of disease (year/never recorded).

The results of the questionnaire were digitalised in a Microsoft Excel table and imported into the R statistical software version 4.0.2. (R Foundation for Statistical Computing, Austria). R-scripts were used to graphically present the disease status and the disease control status (18, 19). If countries had regions with different disease statuses the lowest status was selected as the designated country-level status and used for producing the maps. If a country had only regional CPs, this was sufficient for the country to be regarded as having a CP, for the purposes of producing the maps.

## Results

### Overview of the Control Programmes and Disease Statuses for Each Country

**Figure 2:**
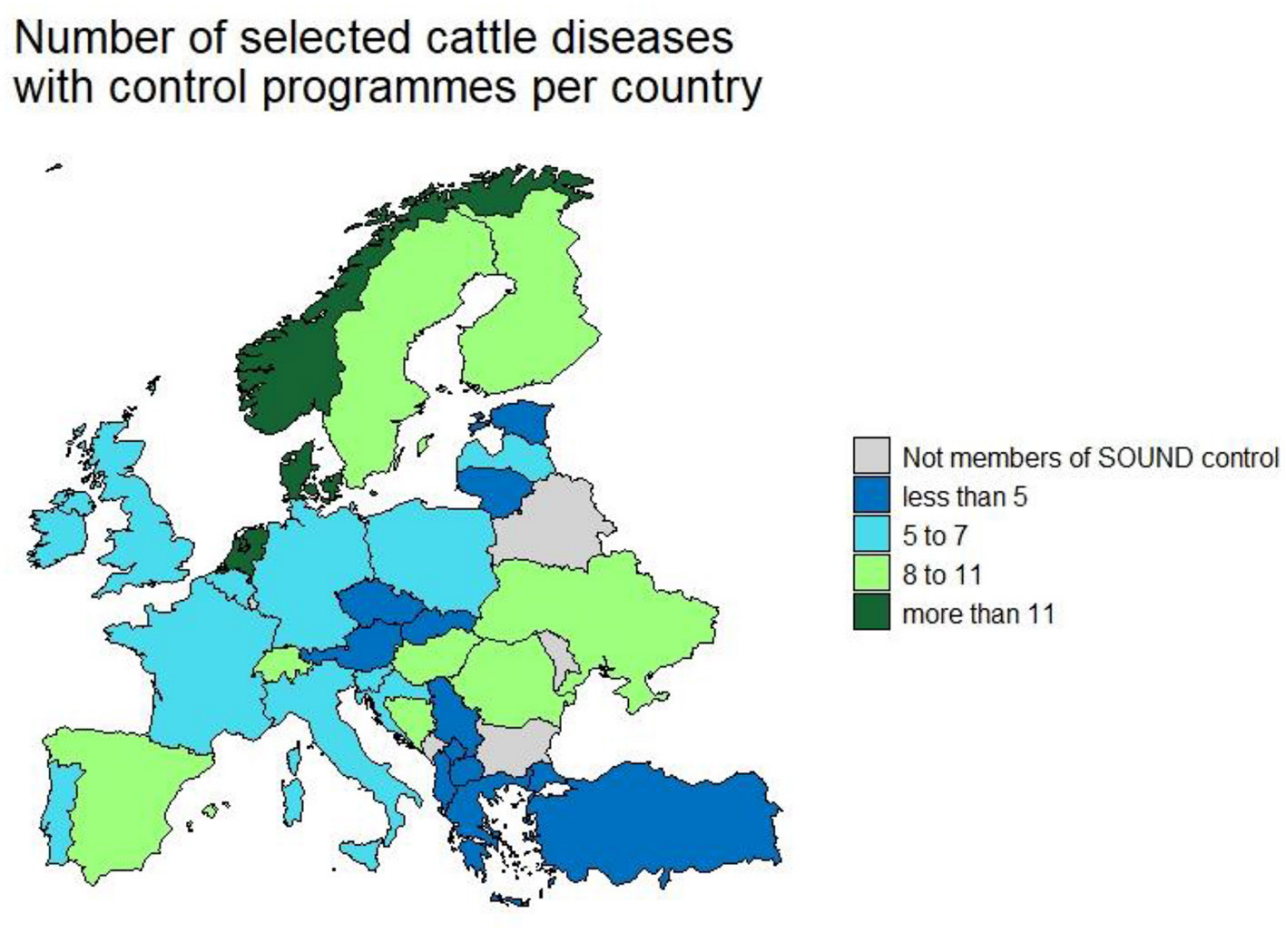
Number of selected cattle disease-free statuses in countries participating in Standardising output-based surveillance to control non-regulated diseases of cattle in the European Union (SOUND control).

In total partners from 33 countries (giving a 100% response rate) provided information (Figure 2). The median number of CPs in place per country was 6 (range 1–13) (Table 2). The number of selected cattle diseases with CPs per country is shown in Figure 2. EBL, BT, IBR, BVD, anthrax, paratuberculosis, salmonellosis, bovine genital campylobacteriosis, leptospirosis and trichomonosis were controlled by the most countries (top 10); therefore, their results will be provided in more detail. Note that throughout the results section percentages may not sum to 100%. This reflects the fact that some countries have not answered all the questions for their CPs in the survey, therefore some information is missing.

**Table 2:** Number of control programmes (CPs) and free statuses per country.

**Country**	**Number of CPs**	**Number of free statuses**
Denmark	13	10
Netherlands	12	9
Norway	12	12
Spain	10	5
Sweden	10	11
Ukraine	10	3
Hungary	10	7
Bosnia and Herzegovina	9	0
Romania	9	0
Finland	8	10
Switzerland	8	4
France	7	1
Ireland	7	7
Belgium	6	5
Germany	6	6
Poland	6	5
Portugal	6	3
UK	6	6
Italy	6	5
Croatia	5	4
Latvia	5	9
Slovenia	5	7
Austria	4	9
Serbia	4	2
Turkey	4	0
Estonia	3	8
Kosovo	3	0
Slovakia	3	5
Lithuania	2	5
Czech Republic	2	6
Albania	1	0
Greece	1	0
Macedonia	1	0

**Figure 3:**
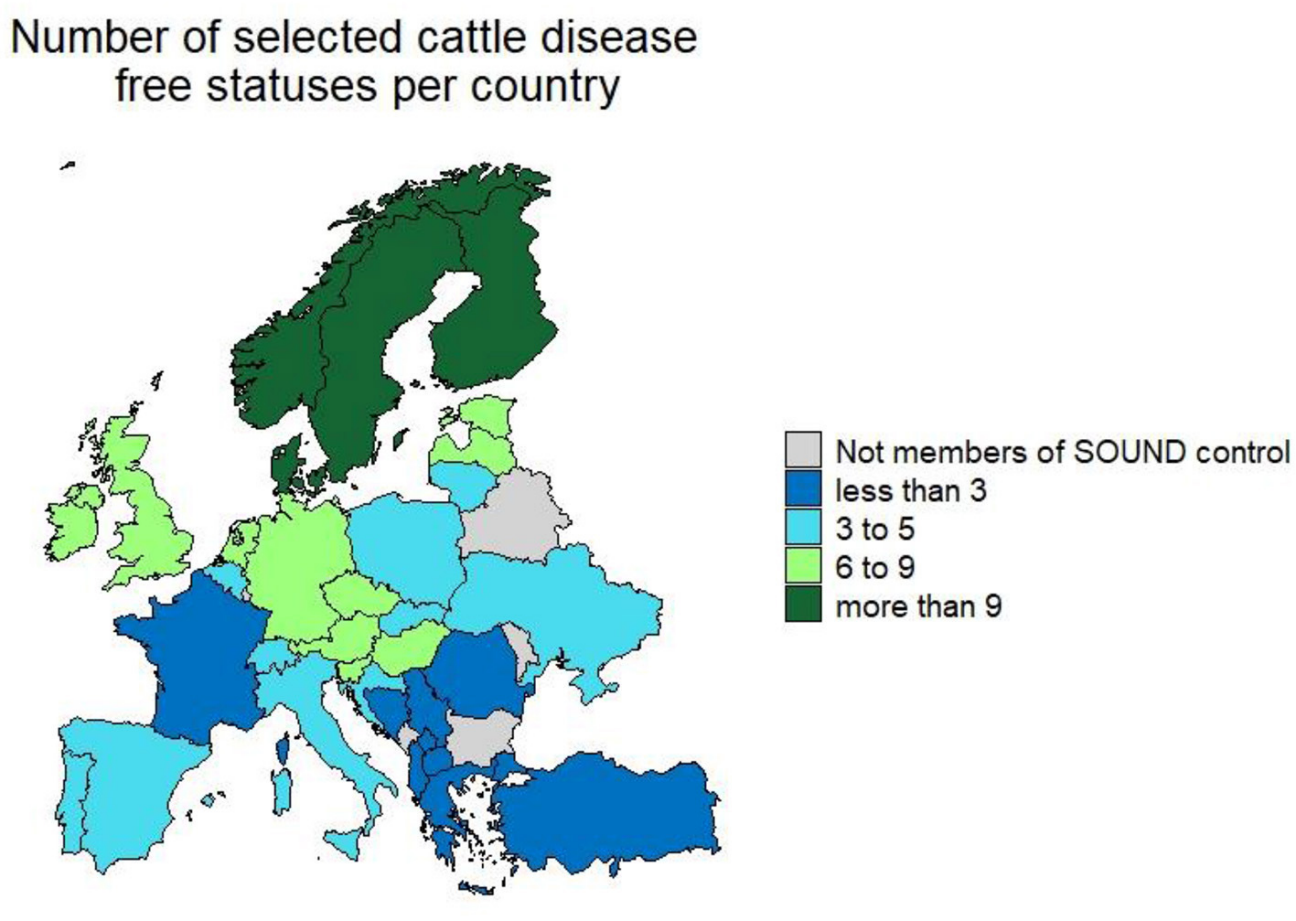
Number of selected cattle diseases with control programmes in countries participating in Standardising output-based surveillance to control non-regulated diseases of cattle in the European Union (SOUND control).

EBL was the most controlled disease (CPs in 31 countries) and the most countries were officially free or perceived free of EBL (22 countries). The country, with CPs for the greatest number of diseases, was Denmark (*n* = 13). Scandinavian countries were free of the most diseases. Norway tops this list, with officially or perceived free status for 12 diseases (Figure 3). Most CPs were implemented at national level (86%) and applied to all types of cattle (75%). The others applied specifically to beef or dairy cattle or breeding animals. Most CPs were compulsory (67%). Most programmes were funded by the government (47%), followed by private (27%) and co-funded programmes (22%). Eradication and control programmes predominate while surveillance programmes are the most common in countries which have eradicated or never had a specific disease and conduct surveillance to prove freedom of disease. Countries that have eradicated diseases like EBL, BT, IBR and BVD have implemented CP for other diseases to further improve the health status of cattle in their country. Austria, Denmark, Finland, Norway and Sweden have an officially free or perceived free status for these four diseases and have on average more CPs in place 9 compared to 6 in countries that are not free. The number of countries with CPs in place per disease are listed in Table 3. The remaining diseases for which CPs were in place in participating countries are presented in Supplementary Material.

**Table 3:** List of selected diseases with a control programme in at least one country participating in the survey and the number of countries with control programmes (CP) per disease.

**No**.	**Cattle disease**	**Number of countries that have a CP in place**
1.	Enzootic Bovine Leukosis (EBL)	31
2.	Bluetongue	27
3.	Infectious Bovine Rhinotracheitis (IBR)	24
4.	Bovine Viral Diarrhoea (BVD)	23
5.	Anthrax	16
6.	Paratuberculosis	15
7.	Salmonellosis	8
8.	Bovine genital campylobacteriosis	7
9.	Leptospirosis	7
10.	Trichomonosis	7
11.	Neosporosis	6
12.	Liver fluke	5
13.	Streptococcal infection	5
14.	Q fever	4
15.	Aujeszky's disease	4
16.	Mycoplasmosis	3
17.	Contagious bovine pleuropneumonia	2
18.	Staphylococcal infection	2
19.	Bovine respiratory disease	2
20.	Epizootic haemorrhagic disease	1
21.	Bovine coronavirus infection	1
22.	Ringworm	1
23.	Bovine digital dermatitis	1

### Overview of the Control Programmes and Disease Statuses for the 10 Diseases of Cattle Most Commonly Subjected to CPs

#### Enzootic Bovine Leukosis

**Figure 4:**
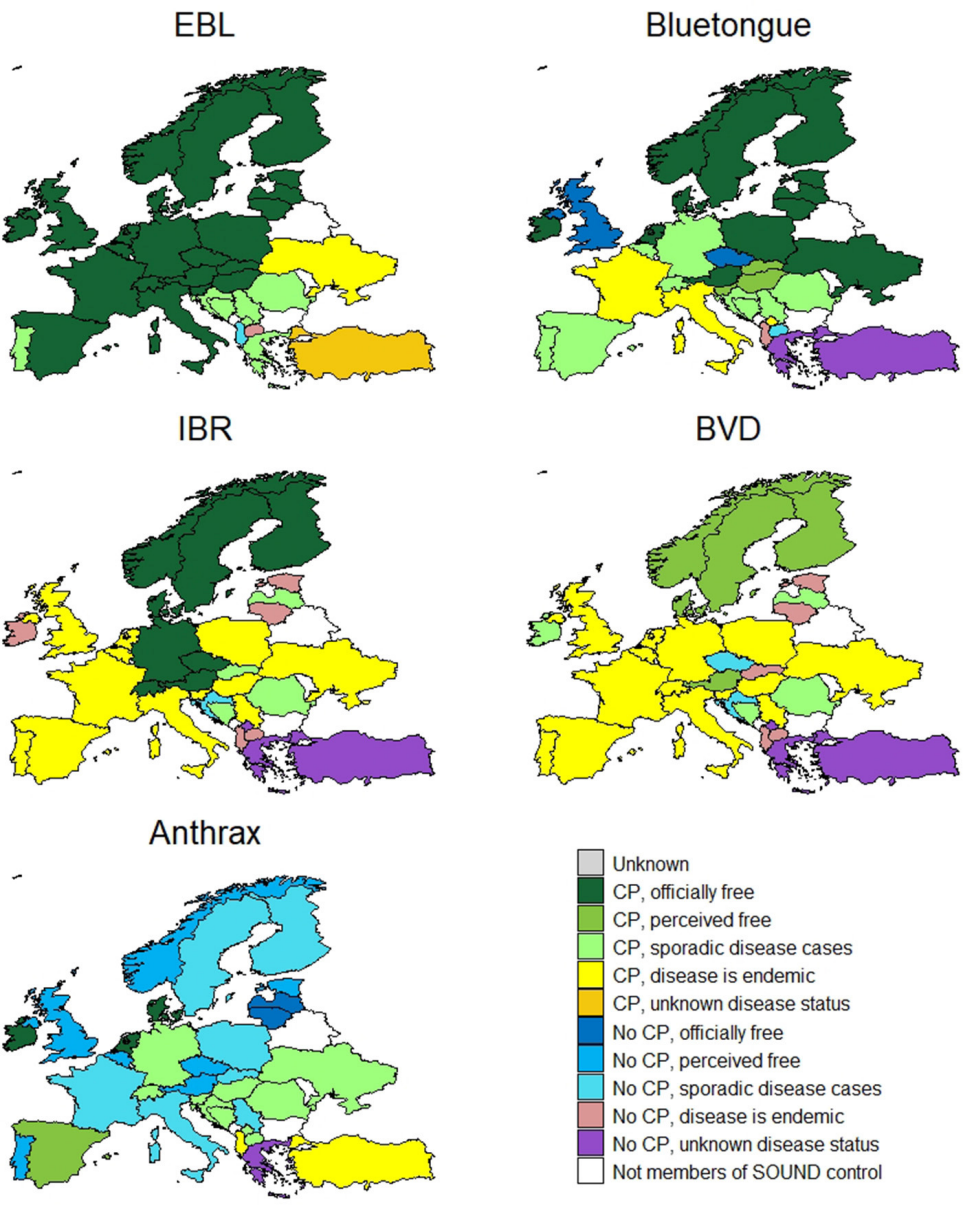
Country level information on control programme implementation and disease status for enzootic bovine leukosis (EBL), bluetongue, infectious bovine rhinotracheitis (IBR), bovine viral diarrhoea (BVD) and anthrax.

The country level information on CP implementation (31 CPs) and disease status for EBL is displayed in Figure 4. Most CPs are applied at national level to all types of cattle and are compulsory (*n* = 28). The vast majority are funded by the government (*n* = 26). The aims of the CPs vary between eradication and surveillance. Twenty-two countries (out of 31 with CPs) are free from the disease and Portugal has most regions free from the disease, except one with sporadic cases. The disease is endemic in two countries, sporadic in eight countries and Turkey does not know its status for the disease.

#### Bluetongue

The country specific information for BT (27 CPs) is displayed in Figure 4. All CPs in place are compulsory (except in Romania which has a voluntary CP) and all are implemented at a national level, mostly government-funded (*n* = 20). The most common type of CPs in place are surveillance programmes (*n* = 16). Seventeen countries are officially free or perceive themselves as free from the disease. Ten countries have a sporadic status, four an endemic status and two countries do not know their status for this disease.

#### Infectious Bovine Rhinotracheitis/Infectious Pustular Vulvovaginitis

The data for IBR/IPV (24 CPs) are presented in Figure 4. Fifteen CPs in place for IBR are compulsory. Most are implemented at a national level (*n* = 19) (Italy, France, Portugal, Spain, and Ukraine have regional CPs). Funding for these comes from a variety of sources [private (43%), government (35%) or co-funded (22%)] and most of the CPs aim to control the disease. The disease is endemic in most of the countries, except for eight that are officially free (eleven have additional EU guarantees for cattle trade). Italy has regions that are officially free of the disease. Five countries have sporadic disease occurrence and three do not know their status for this disease.

#### Bovine Viral Diarrhoea

The country level information for BVD (23 CPs) is displayed in Figure 4. There is a large variety of CPs in place for BVD targeted at breeding animals (9%), dairy cattle (9%) or all types of cattle (82%). Whilst most of the BVD CPs reported are compulsory, a large proportion are voluntary (62:38%). In some countries there is a mixture of compulsory and voluntary CPs depending on region or cattle type e.g., mandatory for dairy and voluntary for non-dairy. The majority of the CPs are implemented at national level (77%) and are privately funded (50%). However, there are also some co-funded programmes (27%) (i.e., funded by government and private stakeholders). The majority of the programmes aim at controlling or eradicating the disease (*n* = 18). Five countries perceive they are free, while for the others the disease occurs sporadically (*n* = 6), is endemic (*n* = 19) or has an unknown status (*n* = 3).

#### Anthrax

The country specific information on CP implementation and disease status for anthrax is displayed in Figure 4. Sixteen countries have a CP. All CPs in place are compulsory and most are implemented at national level (regional in North Macedonia). The majority of CPs are funded by the government (*n* = 11). Thirteen countries are officially free or perceived to be free from the disease. Most remaining countries have sporadic disease cases, while it is endemic in Albania and Turkey. Greece has an unknown disease status.

#### Paratuberculosis

**Figure 5:**
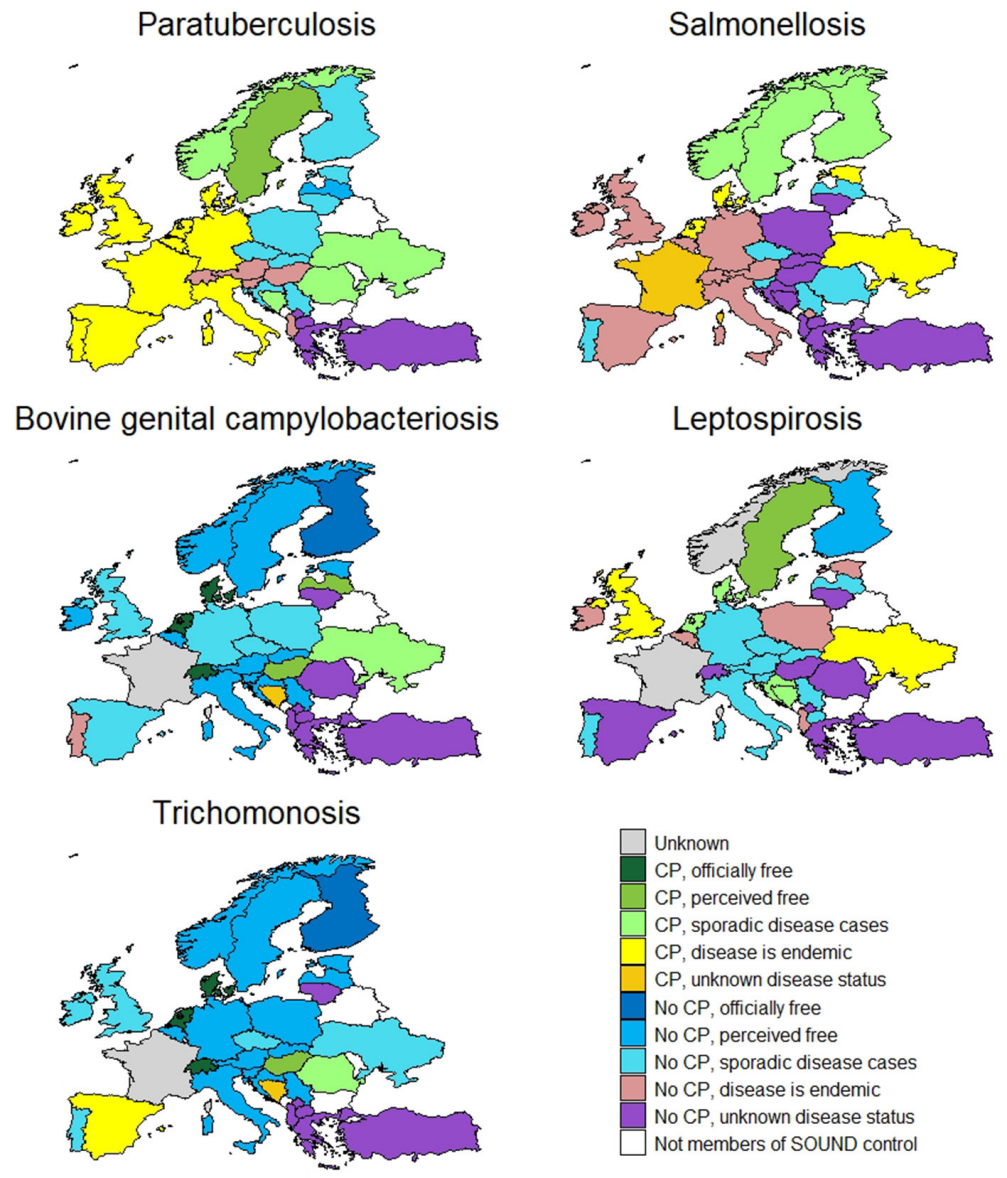
Country level information on control programme implementation and disease status for paratuberculosis, salmonellosis, bovine genital campylobacteriosis, leptospirosis and trichomonosis.

The member countries' information for paratuberculosis (15 CPs) is displayed in Figure 5. Around two thirds of the CPs in place are voluntary (71%). Most apply to all types of cattle, one (Sweden) only applies to beef and four (Belgium, Denmark, Ireland, Netherlands) only to dairy cattle. Bosnia and Herzegovina has a CP for breeding bulls. Sweden has a CP for beef cattle as the country is perceived free and imported beef cattle are considered a risk for disease reintroduction. In the Netherlands non-dairy herds can also participate in a voluntary paratuberculosis CP. Most CPs are implemented at the national level except five (France, Portugal, Spain, Ukraine, and Germany), which are implemented at a regional level. In terms of funding, there is an equal share of programmes privately funded (*n* = 6) and co-funded (*n* = 6), while Germany's and Norway's CP are completely funded by the government. The majority of programmes aim to control the disease (*n* = 9), while four countries have surveillance programmes. Two countries (Latvia and Sweden) are perceived to be free from the disease, twelve have sporadic cases, and four do not know their status. In other countries, the disease is endemic.

#### Salmonellosis

The information on bovine salmonellosis is displayed in Figure 5. Eight countries have a salmonellosis CP in place, of which most are compulsory (*n* = 7). Most are applied to all types of cattle at national level but one (France) is applied at regional level. In the Netherlands the CP for dairy cattle is compulsory, whilst for beef cattle there is a voluntary CP. Funding varies between private (*n* = 1), co-funded (*n* = 3) or government (*n* = 3). Most CPs aim to control and eradicate the disease (*n* = 6). No country is free from the disease, but nine countries report only having sporadic cases and three of those have additional EU guarantees for cattle trade in place (Finland, Norway and Sweden).

#### Bovine Genital Campylobacteriosis

The data for bovine genital campylobacteriosis (7 CPs) are displayed in Figure 5. Most of the countries have national CPs in place (*n* = 6) based on surveillance of breeding bulls, which is not covered under Council Directive 88/407/EEC (16). Most of the CPs are compulsory (*n* = 5). Funding comes from private stakeholders (*n* = 4), government (*n* = 1) or co-funded (*n* = 2) programmes. Seventeen countries are officially free or perceive themselves as free from the disease. Six countries have sporadic cases and the diseases is endemic in Portugal.

#### Leptospirosis

Leptospirosis CPs exist in 7 countries (Figure 5). Most programmes are government-funded. The types of CPs vary between compulsory (*n* = 6) and voluntary (*n* = 2). The Netherlands have a compulsory CP for dairy and a voluntary CP for non-dairy cattle. All CPs are national. Two countries perceive themselves as free (Finland and Sweden). Leptospirosis is endemic in 7 countries, 14 have sporadic cases and 8 do not know their disease status.

#### Trichomonosis

The countries' information for trichomonosis (7 CPs) is displayed in Figure 5. Most of the countries have national compulsory CPs in place based on surveillance of breeding bulls (*n* = 4), which is not covered under Council Directive 88/407/EEC (16). Funding comes from private stakeholders (*n* = 2), government (*n* = 1) or co-funded (*n* = 3) programmes. Eighteen countries are officially free or perceive themselves free from the disease. The disease is endemic in Spain and has a sporadic occurrence in 6 countries.

## Discussion

The aim of this survey was to provide an overview of the control efforts and the disease status of selected cattle diseases with no or limited EU regulation in place, but which are being controlled in at least one European country. At a preliminary evaluation, 23 cattle diseases met the set criteria and were included for further exploration of the status and control efforts in the 33 participating European countries.

Most of the participating countries have a CP for EBL, IBR, BVD, BT and anthrax, while other diseases are controlled by only a few or just a single country. Countries that have eradicated diseases like enzootic bovine leukosis, infectious bovine rhinotracheitis and bovine viral diarrhoea have implemented CPs for other diseases to further improve the health status of cattle in their country.

The highest attainable health status for BVD and paratuberculosis was the perceived free status as there is currently no free status officially recognised by the EU.

The selected diseases of cattle in this survey were defined as those that are not included in either category A or B of the European AHL. Generally, the categorisation C to E in the AHL excludes exotic diseases in the EU and diseases that the EU aims to control with the goal to eradicate. Nevertheless, diseases like bluetongue and EBL are not included in categories A or B, but are subjected to some control by the EU as a number of measures that have to be implemented in EU member states to facilitate trade within the EU are prescribed. These measures are written in directives [EBL: 64/432/EEC (20); BT: 2000/75/EC (21), 2012/5/EU (22)]. Given that they were not categorised as A or B in the AHL both diseases were kept on the list of selected cattle diseases to evaluate the between country differences, as some countries are not part of the EU. Nevertheless, the fact that there is still some regulation in place likely results in many countries implementing some level of control for these diseases, which logically results in a top ten placement of most controlled diseases that are not categorised as A or B in the AHL.

IBR also has a directive describing the sampling protocol for the acquisition and maintenance of farm free statuses [2004/558/EC (23)]. The directive provides a list of free countries and countries which have an EU approved eradication programme. On the basis of this list, countries can ask for additional EU guarantees for animal trade. Requirements for approved CP (3 responding countries) and officially free status (7 responding countries) are input-based. However, countries can also decide to implement their own CPs, even though these are not acknowledged by the EU, as has been done by 15 of the responding countries. The reason for implementing a non-acknowledged programme were related to controlling the losses associated with IBR in the countries/cattle herds or to altogether eradicate IBR in the country. Voluntary programmes are not acknowledged by the EU; however, they can be beneficial to the situation in the country. Because the requirements of the acknowledged CP are not cost-effective, the Netherlands have implemented a national CP that does not meet the EU standards but will reduce the IBR prevalence and eliminate the disease. The approval of output-based standards of such programmes would be very helpful in this regard (24).

The directives of some diseases (e.g., EBL, BT, IBR) were repealed by the AHL 2016/429/EU (15) on April 21, 2021. There are new commission delegated/implementing regulations 2020/687/EU (25), 2020/688/EU (26), 2020/689/EU (27), and 2020/690/EU (28) describing the rules for transport and surveillance within the EU.

For diseases like EBL, BT and IBR that have officially recognisable disease-free statuses, the survey results were compared with EU Commission Decisions. For EBL, compared to the list of countries in Chapter 1 of Annex III to Decision 2003/467/EC (29) with all its amendments, the statuses are comparable apart from Romania (officially free). Despite the officially free status Romania still has sporadic disease cases in the Danube Delta. According to the table with information on the restricted zones for a specific bluetongue serotype or combination of serotypes in accordance with Article 2 (d) of Commission Regulation (EC) No 1266/2007,^1^ restriction zones are still in place in 15 member states: France, Italy, Malta, Croatia, Spain, Portugal, Greece, Bulgaria, Romania, Hungary, Slovenia, Cyprus, Belgium, Germany, Luxembourg. For IBR, compared to Commission Decision 2004/558/EC (23), the responses from member countries match. Belgium, and regions of Italy and France have approved eradication programmes.

The categorisation of diseases by the European commission for the AHL depended on the presence of the disease in the EU, the transmissibility, the routes of transmission, the number of species it affects, the morbidity and mortality, the zoonotic risk, ease of diagnosis and treatment, the economic impact, and the effect on biodiversity, the environment and animal welfare (15). In general, these factors are also taken into account when designing a CP on regional or country level. For example, when country-level disease prevalence is high, the approach to eradication will differ from that for a disease that occurs only sporadically. Factors that play an important role in determining whether to implement a CP include significant economic losses associated with the disease or zoonotic potential (30). Other factors also include the contribution of the cattle industry to the gross domestic product and the predominant cattle production system. Countries with a strong cattle industry and export of live cattle and their products are more motivated to increase cattle production and the quality of their products by controlling infectious diseases. Implementation also depends on cohesive private-public-partnership which is preferred for the functioning of successful CPs. Depending on country and disease, there can be two approaches to CP implementation: bottom-up or top-down initiatives. A bottom-up initiative for disease control (e.g., by farmers and veterinarians striving for coordinated effort on a national or regional level) can start on a voluntary or mandatory basis. Often these CPs start with a small group of farmers or a simple range of initial activities and become stricter over time. Conversely, in a top-down initiative the government requires disease control interventions to be implemented by farmers. Many CPs are a combination of the two (30). The epidemiological characteristics of the pathogen also influence the implementation of a CP. For example, the presence of a specific *Culicoides* spp. vector that is known to be capable to transmit bluetongue in a region or country affects the implementation and design of bluetongue CPs. Epidemiological characteristics also influence disease control strategies. For control of BVD, the strategy can rely specifically on testing for virus presence combined with animal movement restrictions, as the virus does not survive for long in the environment. However, biosecurity is still important as BVD can be transmitted via fomites. For pathogens like *Salmonella* spp. and *Mycobacterium avium* subsp. *paratuberculosis* which can survive for longer outside the host, the CPs must focus on implementation of additional biosecurity measures to prevent or reduce the possibility of direct and indirect transmission through fomites and the environment (30). Out of the 23 diseases for which a CP exists in at least one country in this survey 8 were viral, 11 were bacterial, 3 were parasitic and one was fungal in aetiology.

Even with the predefined definitions the acquisition of the disease statuses was still difficult, as there is no strict cut-off value that divided some of the statuses, e.g., between sporadic and endemic, and sporadic and perceived free. For example, the Commission Decision 2003/467/EC (29) states that free status can be obtained when <0.2% of herds are infected with EBL. In this case a country can be officially free and still have sporadic disease cases. Therefore, the disease statuses may be classified differently between countries despite their having a similar number of cases. It depends on how strict the members were when evaluating their country's data.

The control of anthrax was also debated as only passive surveillance can be organised to detect cases due to the peracute nature of the disease. Also, *Bacillus anthracis* spores can remain in the ground for many years (31). Therefore, if a country had more than just reactive measures for specific outbreaks in place (e.g., movement restrictions, disposal of carcases and disinfection), such as a CP based on vaccination of animals at-risk or on other long-term control measures, they were considered as having a CP.

The limitation of this survey was that it provided only a snapshot of the disease statuses and control programmes in Europe for a specific time frame (end of 2020). Disease statuses and CPs continuously change and the results may become outdated in due course. Therefore, the members of SOUND control have decided to update the information on the SOUND control website^2^ until the end of the action in 2022. The survey also did not cover the whole of Europe. The data for a few countries were not collected because there were no members in SOUND control from these countries. However, a great majority of the European countries were represented and we do not expect the additional information would influence the results much. The fact that these countries do not participate in this COST action may indicate that they are not focussed on these selected cattle diseases. Other limitations of this survey are that the information was provided by members themselves, often including a group of experts with different interpretation of the definitions or the information that was requested from them. This issue was addressed by organizing a series of workshops and discussions to align and agree the definitions. Gathering the information was challenging because of data heterogeneity and the number of countries and experts involved. In some instances, countries did not know their status for certain diseases because they do not test for the disease. In countries where private companies run the CPs the information was not readily available. Where only regional CPs are in place there is often no centralised information system which would allow easy access to this information. Therefore, some of the disease status information was completed using expert opinion or unpublished monitoring results. In the case of France, which has many regional CPs with no centralised database, the members were not confident in reporting information they were not sure of. Because the survey used specific definitions there was no readily available independent information source by which to confirm or compare the data that were provided.

The control and prevalence of cattle diseases in Europe is very heterogeneous and warrants further research. The next step is to collect more information on detailed aspects of the CPs. Therefore, efforts have been made to compile a special issue publication dedicated to describing the control of cattle diseases in each country in a more detailed way.

## Conclusion

This survey provides an overview of CPs in place and cattle disease statuses in European countries, which could be useful for farmers and veterinary authorities when evaluating the risks associated with importing live cattle from the studied countries. The control selected cattle disease is very heterogeneous due to the wide variation in disease prevalence and the corresponding variation in CP design resulting from the need for each country's CP to be tailored to its specific disease context. This warrants a move towards the use of output-based standards for between-country comparison of the statuses resulting from these CPs. Although there is high heterogeneity in CPs, we believe that outcome-based comparison is possible given that each CP developed for a specific disease focuses on control of the same epidemiological characteristics, albeit the dynamics of disease may vary substantially according to factors such as the climate and topography of the country/region affected. The next step in the SOUND control action is to collect more information on detailed aspects of the CPs, which would allow their comparison in a more standardised way.

## Data Availability Statement

The raw data supporting the conclusions of this article will be made available by the authors, without undue reservation.

## Author Contributions

All authors contributed to the study and the writing of this manuscript.

## Conflict of Interest

The authors declare that the research was conducted in the absence of any commercial or financial relationships that could be construed as a potential conflict of interest.

## Publisher's Note

All claims expressed in this article are solely those of the authors and do not necessarily represent those of their affiliated organizations, or those of the publisher, the editors and the reviewers. Any product that may be evaluated in this article, or claim that may be made by its manufacturer, is not guaranteed or endorsed by the publisher.

## Footnotes

^1^ https://ec.europa.eu/food/sites/food/files/animals/docs/ad_control-measures_bt_restrictedzones.pdf

^2^ https://sound-control.eu/
