# Corrigendum: Overview of Cattle Diseases Listed Under Category C, D or E in the Animal Health Law for Which Control Programmes Are in Place Within Europe

**DOI:** 10.3389/fvets.2022.902559

**Published:** 2022-04-20

**Authors:** Jaka Jakob Hodnik, Žaklin Acinger-Rogić, Mentor Alishani, Tiina Autio, Ana Balseiro, John Berezowski, Luís Pedro Carmo, Ilias Chaligiannis, Beate Conrady, Lina Costa, Iskra Cvetkovikj, Ivana Davidov, Marc Dispas, Igor Djadjovski, Elsa Leclerc Duarte, Céline Faverjon, Christine Fourichon, Jenny Frössling, Anton Gerilovych, Jörn Gethmann, Jacinto Gomes, David Graham, Maria Guelbenzu, George J. Gunn, Madeleine K. Henry, Petter Hopp, Hans Houe, Elena Irimia, Jožica Ježek, Ramon A. Juste, Emmanouil Kalaitzakis, Jasmeet Kaler, Selcuk Kaplan, Polychronis Kostoulas, Kaspars Kovalenko, Nada Kneževič, Tanja Knific, Xhelil Koleci, Aurélien Madouasse, Alvydas Malakauskas, Rene Mandelik, Eleftherios Meletis, Madalina Mincu, Kerli Mõtus, Violeta Muñoz-Gómez, Mihaela Niculae, Jelena Nikitović, Matjaž Ocepek, Marie Tangen-Opsal, László Ózsvári, Dimitrios Papadopoulos, Theofilos Papadopoulos, Sinikka Pelkonen, Miroslaw Pawel Polak, Nicola Pozzato, Eglé Rapaliuté, Stefaan Ribbens, João Niza-Ribeiro, Franz-Ferdinand Roch, Liza Rosenbaum Nielsen, Jose Luis Saez, Søren Saxmose Nielsen, Gerdien van Schaik, Ebba Schwan, Blagica Sekovska, Jože Starič, Sam Strain, Petr Šatran, Sabina Šerić-Haračić, Lena-Mari Tamminen, Hans-Hermann Thulke, Ivan Toplak, Erja Tuunainen, Sharon Verner, Štefan Vilček, Ramazan Yildiz, Inge M. G. A. Santman-Berends

**Affiliations:** ^1^Clinic for Reproduction and Large Animals – Section for Ruminants, Veterinary Faculty, University of Ljubljana, Ljubljana, Slovenia; ^2^Veterinary and Food Safety Directorate, Ministry of Agriculture, Zagreb, Croatia; ^3^Department of Veterinary Medicine, Faculty of Agriculture and Veterinary, University of Prishtina “Hasan Prishtina”, Prishtina, Albania; ^4^Finnish Food Authority, Veterinary Bacteriology and Pathology Unit, Kuopio, Finland; ^5^Animal Health Department, University of León, León, Spain; ^6^Animal Health Department, Instituto de Ganadería de Montaña Consejo Superior de Investigaciones Científicas-University of León, León, Spain; ^7^Veterinary Public Health Institute, Vetsuisse, University of Bern, Bern, Switzerland; ^8^School of Veterinary Medicine, Aristotle University of Thessaloniki, Thessaloniki, Greece; ^9^Department of Veterinary and Animal Sciences, Faculty of Health and Medical Sciences, University of Copenhagen, Copenhagen, Denmark; ^10^Complexity Science Hub Vienna, Vienna, Austria; ^11^Department of Agrarian and Veterinary Sciences, Agrarian School of Elvas, Polytechnic Institute of Portalegre, Portalegre, Portugal; ^12^Faculty of Veterinary Medicine in Skopje, Ss Cyril and Methodius University in Skopje, Skopje, Macedonia; ^13^Faculty of Agriculture, University of Novi Sad, Novi Sad, Serbia; ^14^Sciensano, Brussels, Belgium; ^15^Departamento de Medicina Veterinária, Mediterranean Institute for Agriculture, Environment and Development, Universidade de Évora, Évora, Portugal; ^16^Ausvet Europe, Lyon, France; ^17^INRAE, Oniris, BIOEPAR, Nantes, France; ^18^Department of Disease Control and Epidemiology, National Veterinary Institute (SVA), Uppsala, Sweden; ^19^Department of Animal Environment and Health, Swedish University of Agricultural Sciences, Skara, Sweden; ^20^National Scientific Centre, Institute for Experimental and Clinical Veterinary Medicine, Kharkiv, Ukraine; ^21^Friedrich-Loeffler-Institut, Federal Research Institute for Animal Health, Institute of Epidemiology, Greifswald, Germany; ^22^Animal Health and Production Unit, National Institute for Agrarian and Veterinary Research, Oeiras, Portugal; ^23^Animal Health Ireland, Carrick on Shannon, Ireland; ^24^Epidemiology Research Unit, Department of Veterinary and Animal Science, Northern Faculty, Scotland's Rural College, Inverness, United Kingdom; ^25^Section of Epidemiology, Norwegian Veterinary Institute (NVI), Oslo, Norway; ^26^Research and Development Institute for Bovine Balotesti, Balotesti, Romania; ^27^Department of Animal Health, NEIKER-Basque Institute for Agricultural Research and Development, Basque Research and Technology Alliance, Derio, Spain; ^28^Clinic of Farm Animals, Veterinary Faculty, Aristotle University Thessaloniki, Thessaloniki, Greece; ^29^School of Veterinary Medicine and Science, University of Nottingham, Nottingham, United Kingdom; ^30^Department of Genetics, Faculty of Veterinary Medicine, Tekirdag Namik Kemal University, Tekirdag, Turkey; ^31^Laboratory of Epidemiology, Faculty of Public and One (Integrated) Health, School of Health Sciences, University of Thessaly, Karditsa, Greece; ^32^Faculty of Veterinary Medicine, Latvia University of Lifesciences and Technologies, Jelgava, Latvia; ^33^Podravka Food Industry, Research and Development, Koprivnica, Croatia; ^34^Veterinary Faculty, Institute of Food Safety, Feed and Environment, University of Ljubljana, Ljubljana, Slovenia; ^35^Department of Veterinary Public Health, Faculty of Veterinary Medicine, Agricultural University of Tirana, Tirana, Albania; ^36^Department of Veterinary Pathobiology, Lithuanian University of Health Sciences, Veterinary Academy, Kaunas, Lithuania; ^37^Department of Epizootiology, Parasitology and Protection of One Health, University of Veterinary Medicine and Pharmacy, Kosice, Slovakia; ^38^Institute of Veterinary Medicine and Animal Sciences, Estonian University of Life Sciences, Tartu, Estonia; ^39^Section of Epidemiology, Vetsuisse Faculty, University of Zürich, Zurich, Switzerland; ^40^Faculty of Veterinary Medicine, University of Agricultural Sciences and Veterinary Medicine Cluj-Napoca, Cluj-Napoca, Romania; ^41^Institute for Genetic Resources, University of Banja Luka, Banja Luka, Bosnia and Herzegovina; ^42^Veterinary Faculty, National Veterinary Institute, University of Ljubljana, Ljubljana, Slovenia; ^43^Norwegian Food Safety Authority, Oslo, Norway; ^44^Department of Veterinary Forensics and Economics, University of Veterinary Medicine Budapest, Budapest, Hungary; ^45^Department of Microbiology, Faculty of Veterinary Medicine, Aristoteles University of Thessaloniki, Thessaloniki, Greece; ^46^National Veterinary Research Institute, Pulawy, Poland; ^47^Laboratorio di Medicina Forense Veterinaria, Struttura Complessa Territoriale 1 - Verona e Vicenza, Istituto Zooprofilattico Sperimentale Delle Venezie, Vicenza, Italy; ^48^Animal Health Care Flanders, Torhout, Belgium; ^49^Department of Population Studies, Institute of Biomedical Sciences Abel Salazar, University of Porto, Porto, Portugal; ^50^Unit of Food Microbiology, Institute for Food Safety, Food Technology and Veterinary Public Health, University of Veterinary Medicine Vienna, Vienna, Austria; ^51^Ministry of Agriculture, Fisheries and Food, Madrid, Spain; ^52^Department of Population Health Sciences, Faculty of Veterinary Medicine, Utrecht University, Utrecht, Netherlands; ^53^Royal GD, Deventer, Netherlands; ^54^Farm and Animal Health, Uppsala, Sweden; ^55^Animal Health and Welfare Northern Ireland, Dungannon, United Kingdom; ^56^State Veterinary Administration, Prague, Czechia; ^57^Animal Health Economics Department, Veterinary Faculty of the University of Sarajevo, Sarajevo, Bosnia and Herzegovina; ^58^Swedish University of Agricultural Sciences, Uppsala, Sweden; ^59^Department of Ecological Modelling, Helmholtz Centre for Environmental Research – UFZ, Leipzig, Germany; ^60^Department of Virology, Veterinary Faculty, Institute of Microbiology and Parasitology, University of Ljubljana, Ljubljana, Slovenia; ^61^Animal Health ETT, Seinäjoki, Finland; ^62^Department of Internal Medicine, Faculty of Veterinary Medicine, Burdur Mehmet Akif Ersoy University, Burdur, Turkey

**Keywords:** disease control, SOUND control, control programmes, Europe, cattle, output-based standards

In the original article, there was an error. We used the phrase “non-regulated” for cattle diseases that are in fact listed in the New Animal Health Law that went into force in 2021.

A correction has been made to **Abstract**. The corrected section is shown below.

The COST action “Standardising output-based surveillance to control non-regulated diseases of cattle in the European Union (SOUND control),” aims to harmonise the results of surveillance and control programmes (CPs) for selected cattle diseases to facilitate safe trade and improve overall control of cattle infectious diseases. In this paper we aimed to provide an overview on the diversity of control for these diseases in Europe. A selected cattle disease was defined as an infectious disease of cattle with no or limited control at EU level, which is not included in the European Union Animal health law Categories A or B under Commission Implementing Regulation (EU) 2020/2002. A CP was defined as surveillance and/or intervention strategies designed to lower the incidence, prevalence, mortality or prove freedom from a specific disease in a region or country. Passive surveillance, and active surveillance of breeding bulls under Council Directive 88/407/EEC were not considered as CPs. A questionnaire was designed to obtain country-specific information about CPs for each disease. Animal health experts from 33 European countries completed the questionnaire. Overall, there are 23 diseases for which a CP exists in one or more of the countries studied. The diseases for which CPs exist in the highest number of countries are enzootic bovine leukosis, bluetongue, infectious bovine rhinotracheitis, bovine viral diarrhoea and anthrax (CPs reported by between 16 and 31 countries). Every participating country has on average, 6 CPs (min–max: 1–13) in place. Most programmes are implemented at a national level (86%) and are applied to both dairy and non-dairy cattle (75%). Approximately one-third of the CPs are voluntary, and the funding structure is divided between government and private resources. Countries that have eradicated diseases like enzootic bovine leukosis, bluetongue, infectious bovine rhinotracheitis and bovine viral diarrhoea have implemented CPs for other diseases to further improve the health status of cattle in their country. The control of the selected cattle diseases is very heterogenous in Europe. Therefore, the standardising of the outputs of these programmes to enable comparison represents a challenge.

A correction has also been made to **Introduction**, Paragraphs 2, 3, 4, and 5. The corrected paragraphs are shown below.

The control of some cattle diseases in the European Union (EU) is currently founded on input-based standards, by which the EU prescribes all the activities a country must implement to reach the desired output, confidence of freedom from infection or disease. However, there is an international trend to move to output-based standards, which do not prescribe how the end goal (confidence of freedom from infection or disease) must be achieved and allows for country specific control or eradication measures (2). The move to output-based standards would allow for safe trade of cattle between territories that have achieved the desired confidence of freedom, without additional costs for testing of individual animals (3). Additionally, because EU member states are not allowed to set trade restrictions on intracommunity trade for some selected cattle diseases, countries that have achieved freedom from specific diseases are at risk of their reintroduction with imported animals. Therefore, available information on the current control and disease status in each country would greatly aid farmers and authorities when considering the risk of importing live cattle from these countries.

“Standardising output-based surveillance to control non-regulated diseases of cattle in the European Union” (SOUND control) is a COST action (CA 17110) aiming to harmonise the results of surveillance and control programmes for selected cattle diseases to facilitate safe trade, and to reduce the economic impact and improve overall control of infectious cattle diseases. This COST action connects more than 100 members from different fields (including veterinarians, epidemiologists, economists, statisticians, sociologists and policy makers) from 33 European countries. An overview of the project was published by Costa et al. (1). The first working group within the action aims to identify cattle diseases with no or limited regulation at European level for which CPs are in place and to describe the characteristics of these CPs. To obtain this information clear definitions of CPs and disease statuses had to be set to allow the comparisons of the heterogeneous CPs.

Similar evaluations have been undertaken for bovine viral diarrhoea and paratuberculosis (3, 4), but these studies were limited to only one disease. In 2017, the European Food Safety Authority (EFSA) published information on EU countries' disease statuses for certain cattle diseases (5–14); however, different definitions were used and not all of the selected diseases were covered. Furthermore, not all European countries were included and some of the data are now outdated.

This paper aims to provide a comprehensive overview of the current (end of 2020) disease status and control efforts for selected cattle diseases with no or limited regulation at European level, for all 33 European countries that participate in the SOUND control project in 2020. To the best of the authors' knowledge, this is the first overview of cattle disease CPs in Europe incorporating so wide a range of diseases and representing so many countries.

A correction has been made to **Materials and Methods**, “*Definitions*,” Paragraph 1. The corrected paragraph is shown below.

The definitions for the survey were agreed upon at a series of meetings involving members of all countries participating in SOUND control. First, the definition of a selected cattle disease had to be clarified. Initially, such diseases were defined as diseases with no or limited regulation at EU level. However, given the adoption of the new Animal Health Law (AHL) (15), most cattle diseases were categorised at some level and the definition of selected cattle diseases had to be aligned with the changed law. Additionally, definitions had to be determined for a disease CP and a country disease status. The final selected definitions were:

A correction has also been made to **Materials and Methods**, “*Definitions*,” Paragraph 3. The corrected paragraph is shown below.

*Selected cattle diseases* are defined as infectious cattle diseases not included in the AHL category A or B (15), but for which there are CPs in place in the COST action member countries. This definition also includes diseases for which eradication has been achieved and surveillance is ongoing.

A correction has been made to a subtitle in the section **Materials and Methods**. The subtitle “*Development of the Questionnaire on Existing Control Programmes for Non-EU Regulated Cattle Diseases*” should instead be written as “*Development of the Questionnaire on Existing Control Programmes for Selected Cattle Diseases*.”

A correction has been made to **Results**, “*Overview of the Control Programmes and Disease Statuses for Each Country*,” Paragraph 1. The corrected paragraph is shown below.

In total partners from 33 countries (giving a 100% response rate) provided information (Figure 2). The median number of CPs in place per country was 6 (range 1–13) (Table 2). The number of selected cattle diseases with CPs per country is shown in Figure 2. EBL, BT, IBR, BVD, anthrax, paratuberculosis, salmonellosis, bovine genital campylobacteriosis, leptospirosis and trichomonosis were controlled by the most countries (top 10); therefore, their results will be provided in more detail. Note that throughout the results section percentages may not sum to 100%. This reflects the fact that some countries have not answered all the questions for their CPs in the survey, therefore some information is missing.

**Figures 2:**
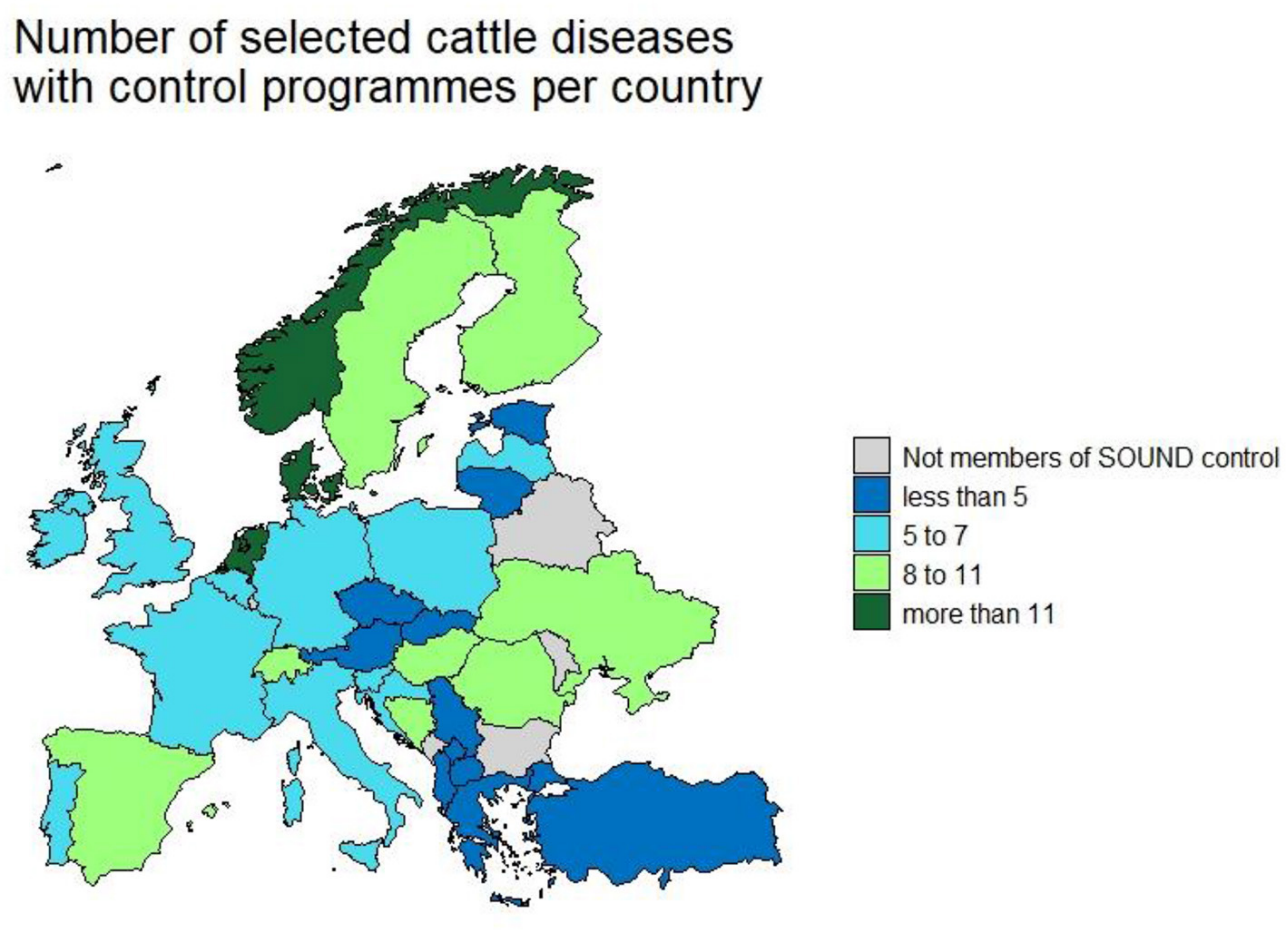
Number of selected cattle disease-free statuses in countries participating in Standardising output-based surveillance to control non-regulated diseases of cattle in the European Union (SOUND control).

Additionally, corrections have been made to several figures and tables, as detailed below.

A correction has also been made to the caption of Figure 2. The corrected caption is shown below.

Figure 2 has also been replaced with an updated version that states “selected cattle diseases” instead of “non-EU regulated cattle diseases.” The updated Figure 2 is shown below.

A correction has also been made to the caption of Figure 3. The corrected caption is shown below.

**Figures 3:**
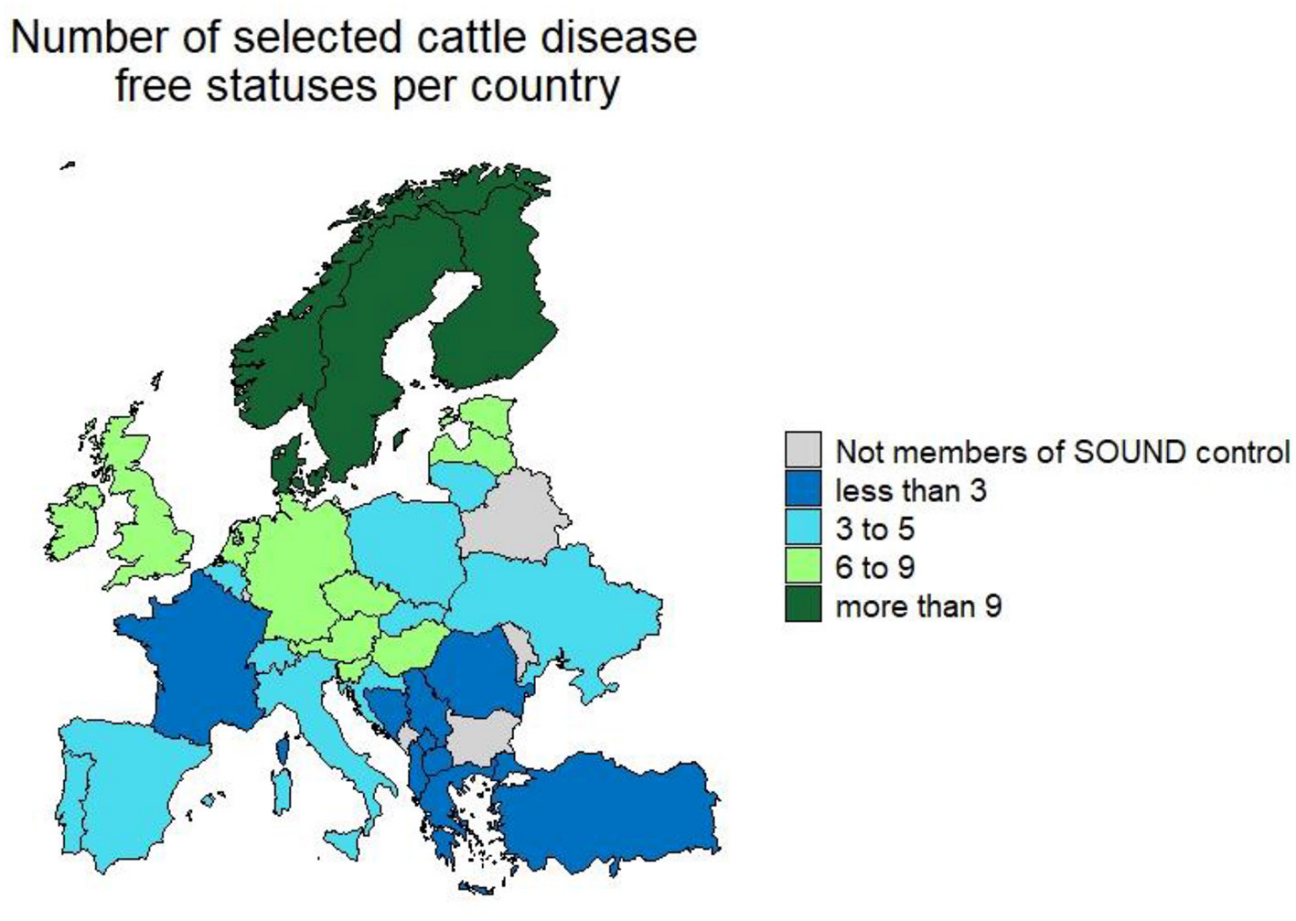
Number of selected cattle diseases with control programmes in countries participating in Standardising output-based surveillance to control non-regulated diseases of cattle in the European Union (SOUND control).

Figure 3 has also been replaced with an updated version that states “selected cattle disease” instead of “non-EU regulated cattle disease.” The updated Figure 3 is shown below.

A correction has been made to the caption of Table 3. The corrected caption is shown below.

Table 3. List of selected diseases with a control programme in at least one country participating in the survey and the number of countries with control programmes (CP) per disease.

Lastly, corrections have been made to **Discussion** and **Conclusion**.

A correction has been made to **Discussion**, Paragraph 1. The corrected paragraph is shown below.

The aim of this survey was to provide an overview of the control efforts and the disease status of selected cattle diseases with no or limited EU regulation in place, but which are being controlled in at least one European country. At a preliminary evaluation, 23 cattle diseases met the set criteria and were included for further exploration of the status and control efforts in the 33 participating European countries.

A correction has been made to **Discussion**, Paragraph 4. The corrected paragraph is shown below.

The selected diseases of cattle in this survey were defined as those that are not included in either category A or B of the European AHL. Generally, the categorisation C to E in the AHL excludes exotic diseases in the EU and diseases that the EU aims to control with the goal to eradicate. Nevertheless, diseases like bluetongue and EBL are not included in categories A or B, but are subjected to some control by the EU as a number of measures that have to be implemented in EU member states to facilitate trade within the EU are prescribed. These measures are written in directives [EBL: 64/432/EEC (20); BT: 2000/75/EC (21), 2012/5/EU (22)]. Given that they were not categorised as A or B in the AHL both diseases were kept on the list of selected cattle diseases to evaluate the between country differences, as some countries are not part of the EU. Nevertheless, the fact that there is still some regulation in place likely results in many countries implementing some level of control for these diseases, which logically results in a top ten placement of most controlled diseases that are not categorised as A or B in the AHL.

A correction has been made to **Discussion**, Paragraph 11. The corrected paragraph is shown below.

The limitation of this survey was that it provided only a snapshot of the disease statuses and control programmes in Europe for a specific time frame (end of 2020). Disease statuses and CPs continuously change and the results may become outdated in due course. Therefore, the members of SOUND control have decided to update the information on the SOUND control website until the end of the action in 2022. The survey also did not cover the whole of Europe. The data for a few countries were not collected because there were no members in SOUND control from these countries. However, a great majority of the European countries were represented and we do not expect the additional information would influence the results much. The fact that these countries do not participate in this COST action may indicate that they are not focussed on these selected cattle diseases. Other limitations of this survey are that the information was provided by members themselves, often including a group of experts with different interpretation of the definitions or the information that was requested from them. This issue was addressed by organizing a series of workshops and discussions to align and agree the definitions. Gathering the information was challenging because of data heterogeneity and the number of countries and experts involved. In some instances, countries did not know their status for certain diseases because they do not test for the disease. In countries where private companies run the CPs the information was not readily available. Where only regional CPs are in place there is often no centralised information system which would allow easy access to this information. Therefore, some of the disease status information was completed using expert opinion or unpublished monitoring results. In the case of France, which has many regional CPs with no centralised database, the members were not confident in reporting information they were not sure of. Because the survey used specific definitions there was no readily available independent information source by which to confirm or compare the data that were provided.

A correction has been made to **Conclusion**. The corrected section is shown below.

This survey provides an overview of CPs in place and cattle disease statuses in European countries, which could be useful for farmers and veterinary authorities when evaluating the risks associated with importing live cattle from the studied countries. The control selected cattle disease is very heterogeneous due to the wide variation in disease prevalence and the corresponding variation in CP design resulting from the need for each country's CP to be tailored to its specific disease context. This warrants a move towards the use of output-based standards for between-country comparison of the statuses resulting from these CPs. Although there is high heterogeneity in CPs, we believe that outcome-based comparison is possible given that each CP developed for a specific disease focuses on control of the same epidemiological characteristics, albeit the dynamics of disease may vary substantially according to factors such as the climate and topography of the country/region affected. The next step in the SOUND control action is to collect more information on detailed aspects of the CPs, which would allow their comparison in a more standardised way.

The authors apologize for these errors and state that they do not change the scientific conclusions of the article in any way. The original article has been updated.

## Publisher's Note

All claims expressed in this article are solely those of the authors and do not necessarily represent those of their affiliated organizations, or those of the publisher, the editors and the reviewers. Any product that may be evaluated in this article, or claim that may be made by its manufacturer, is not guaranteed or endorsed by the publisher.

