# Supplementary material for: Overview of Cattle Diseases Listed Under Category C, D or E in the Animal Health Law for Which Control Programmes Are in Place Within Europe
Source: Front Vet Sci. 2021 Jul 30;8:688078. doi: 10.3389/fvets.2021.688078 (PMC8361752; doi:10.3389/fvets.2021.688078)
Supplement: Supplementary file 1 [file Data_Sheet_1.PDF]

Supplementary document 1

Remaining diseases

Bacterial diseases

Five countries (Denmark, Finland, Italy, Norway and Sweden) have a CP for streptococcal infections (*Streptococcus agalactiae)* (FIGURE 6). Most are national (Italy regional) and voluntary (Italy compulsory). All CPs are privately funded. No country is free of the pathogen. The disease is endemic in most countries and sporadic in Latvia, Slovakia, Slovenia and the Netherlands. Some countries do not know their status.

Four countries have a CP in place for Q-fever (FIGURE 6). The CPs are a mix of compulsory (n=3) and voluntary programmes. The funding varies between the CPs. The disease occurs endemically or sporadically in most countries; although, Norway perceive to be free. Five countries do not know their disease status.

There are CPs for contagious bovine pleuropneumonia *(Mycoplasma mycoides* subsp. *mycoides SC)* in 2 countries (Hungary and Spain) (FIGURE 6). Both programmes are national, compulsory and are funded by the government. Nineteen countries are, or perceive themselves to be, free of the pathogen. Other countries do not know their status.

Three countries have CPs for mycoplasmosis (*Mycoplasma bovis)*. All the CPs are national and privately funded. Norway perceives itself as free of the disease. The pathogen is endemic or the status is unknown for most other countries (FIGURE 6). The Czech Republic, Lithuania and Sweden have sporadic cases.

Two (Switzerland and Ukraine) countries have CPs in place for Staphylococcal infections *(Staphylococcus aureus)*. Norway has a programme only for methicillin-resistant *S. aureus* (MRSA) surveillance in mastitis cases which occur sporadically, *S. aureus* is otherwise endemic in the country. Both programmes are implemented on a regional level. Switzerland has a CP supported by the government, while Ukrainian CP is privately funded. Most countries are endemic (FIGURE 6). Many countries do not know their disease status.

Only Norway has a national, voluntary, privately funded CP for .bovine digital dermatitis. The disease is endemic or the status is unknown (sporadic in the Czech Republic and Slovakia) in most countries (FIGURE 6).

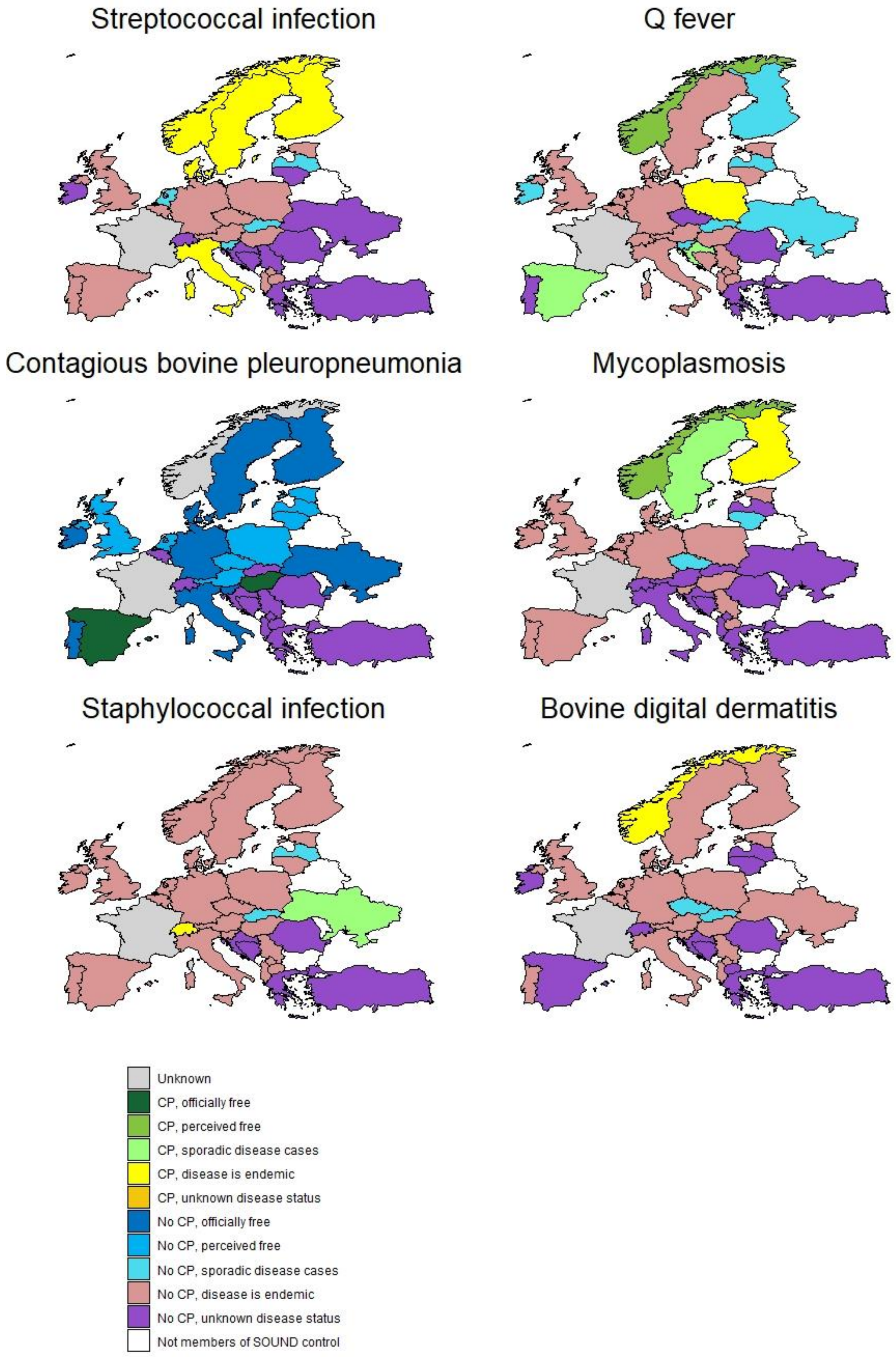
Streptococcal infection
Q fever
Contagious bovine pleuropneumonia
Mycoplasmosis
Staphylococcal infection
Bovine digital dermatitis
Unknown
CP, officially free
CP, perceived free
CP, sporadic disease cases
CP, disease is endemic
CP, unknown disease status
No CP, officially free
No CP, perceived free
No CP, sporadic disease cases
No CP, disease is endemic
No CP, unknown disease status
Not members of SOUND control

FIGURE 6 - Country level information on control programme implementation and disease status for the remaining bacterial diseases.

Viral diseases

The information on CPs and disease status for Aujeszky's disease is displayed in FIGURE 7. Four have CPs in place. All CPs are compulsory (except Romania's) and implemented at a national level. Twenty-one countries are officially free or perceive themselves to be free of Aujeszky's disease. The disease is sporadic in Serbia, Romania and Italy, and 8 countries do not know their status for the disease in cattle.

Epizootic haemorrhagic disease is controlled in Hungary. The CP is based on surveillance. Sixteen countries are or perceive themselves to be free from the disease. Fifteen countries do not know their status (FIGURE 7). The disease is endemic in Switzerland.

Two countries (Norway and Sweden) have a CP for bovine respiratory diseases (bovine respiratory syncytial virus). Both CPs are national and voluntary. The CPs are privately funded. No country is free of the disease. The disease is sporadic in Croatia, the Czech Republic Latvia and Slovakia and endemic or the status is unknown in the other countries (FIGURE 7).

Bovine coronavirus infections are controlled only in Norway. The CP is national, voluntary and privately funded. The disease is endemic or the status is not known in most European countries (sporadic in Serbia, Latvia, North Macedonia, Portugal and Slovakia) (FIGURE 7).

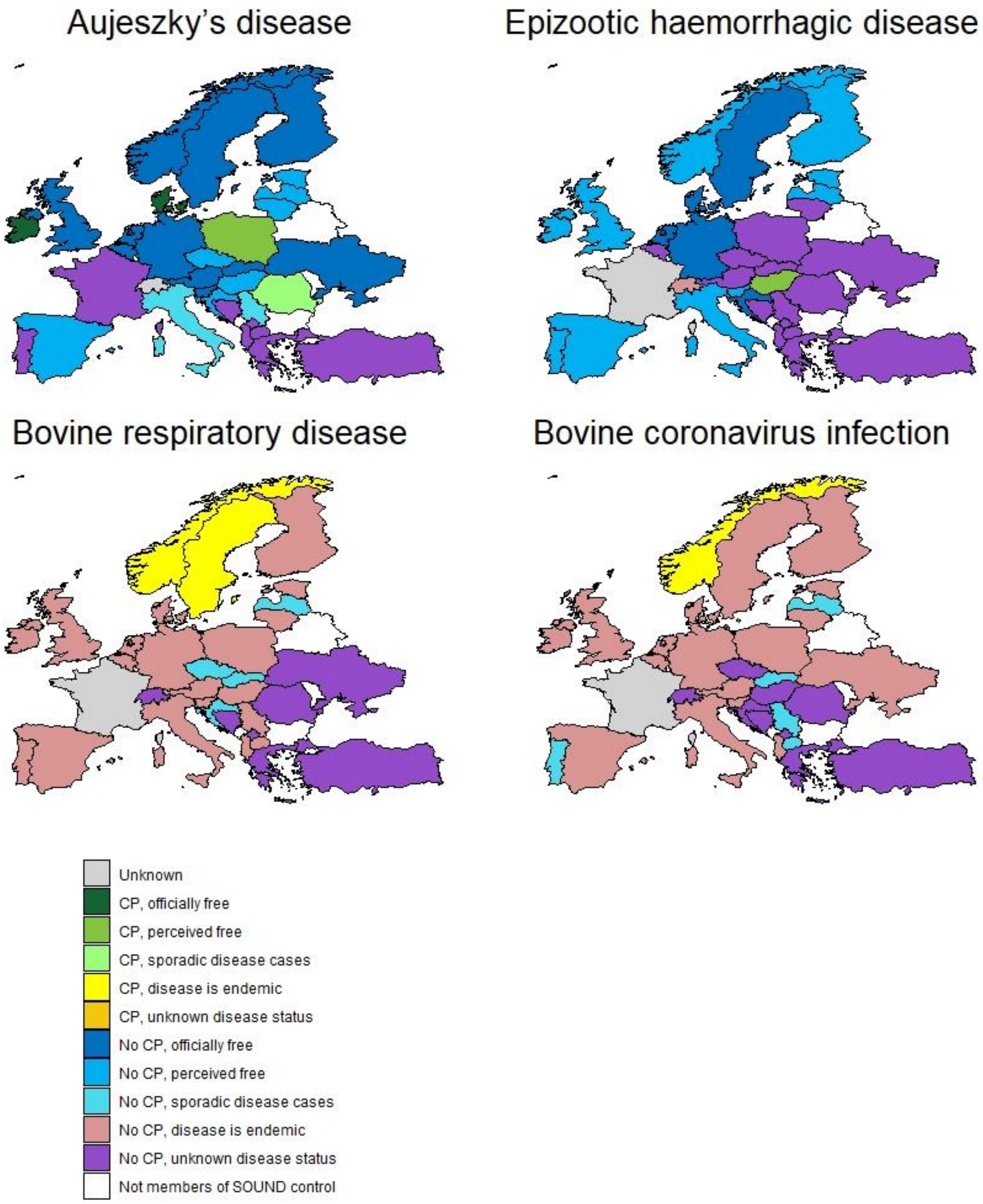

FIGURE 7 - Country level information on control programme implementation and disease status for the remaining viral diseases.

Parasitic, protozoal and fungal diseases

Six countries have CPs in place for neosporosis (FIGURE 8). They are a mix of national (Belgium, the Netherlands and the UK) and regional (France, Portugal and Spain) voluntary programmes. No country declared themselves free from neosporosis. The disease status is endemic, or unknown in most countries (sporadic in Austria, Croatia, Finland, Lithuania and Poland).

Five countries (Denmark, Hungary, Ireland, Netherlands and Romania) have CPs for liver fluke. All CPs are national. Denmark and Hungary have compulsory CPs (meat inspection) funded by the government the remaining are voluntary and privately or co-funded. The disease status is endemic or unknown in most countries (FIGURE 8). Finland, Slovakia and Poland have sporadic disease cases.

Only Finland has a CP for ringworm (*Trichophyton verrucosum)*. The CP is voluntary and privately funded. The disease is endemic in most countries or they do not know their status. Eight countries have sporadic disease cases (FIGURE 8).

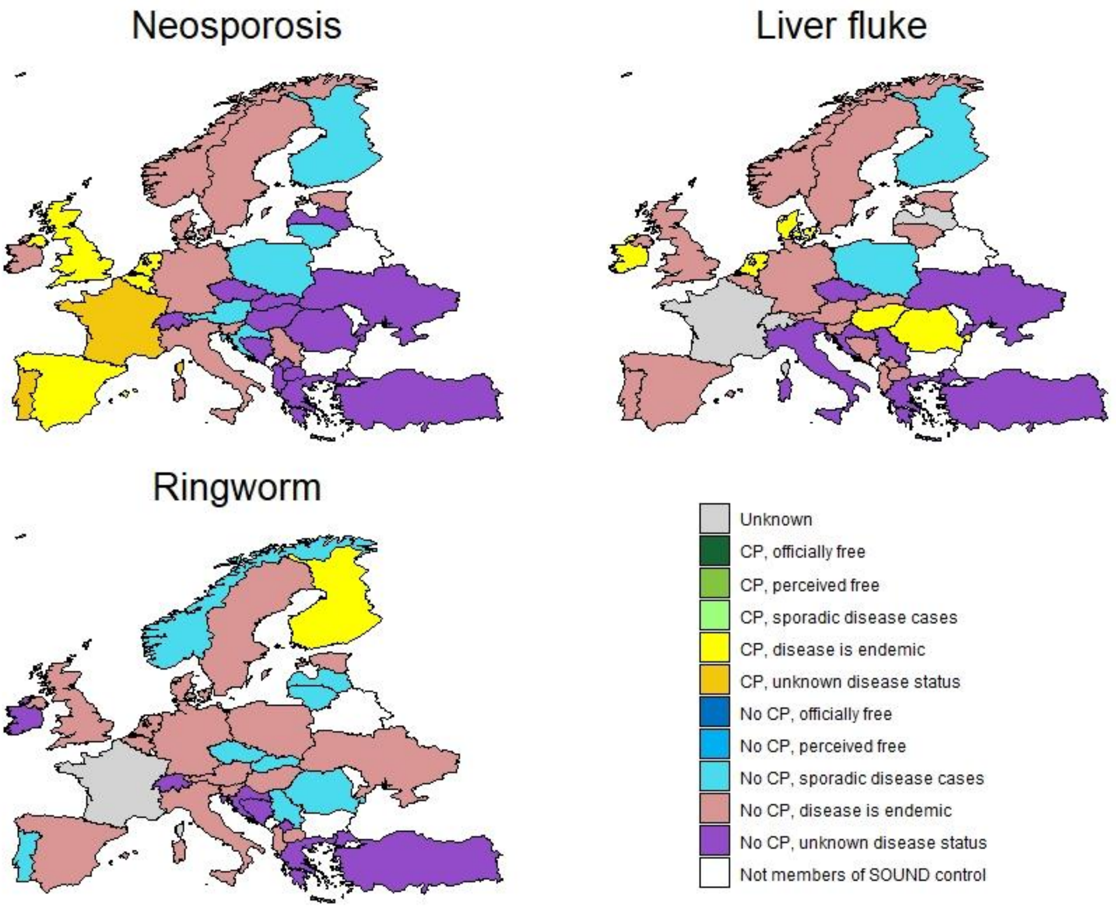

FIGURE 8 - Country level information on control programme implementation and disease status for neosporosis, liver fluke and ringworm.
